# Fragmentation and high entropy of neonatal experience predict adolescent emotional outcome

**DOI:** 10.1038/tp.2015.200

**Published:** 2016-01-05

**Authors:** J Molet, K Heins, X Zhuo, Y T Mei, L Regev, T Z Baram, H Stern

**Affiliations:** 1 Department of Anatomy and Neurobiology, University of California-Irvine, Irvine, CA, USA; 2 Department of Statistics, Donald Bren School of Information and Computer Sciences, University of California-Irvine, Irvine, CA, USA; 3 Department of Pediatrics, University of California-Irvine, Irvine, CA, USA; 4 Department of Neurology, School of Medicine, University of California-Irvine, Irvine, CA, USA

## Abstract

Vulnerability to emotional disorders including depression derives from interactions between genes and environment, especially during sensitive developmental periods. Across evolution, maternal care is a key source of environmental sensory signals to the developing brain, and a vast body of work has linked quantitative and qualitative aspects of maternal care to emotional outcome in children and animals. However, the fundamental properties of maternal signals, that promote advantageous vs pathological outcomes in the offspring, are unknown and have been a topic of intense study. We studied emotional outcomes of adolescent rats reared under routine or impoverished environments, and used mathematical approaches to analyze the nurturing behaviors of the dams. Unexpectedly, whereas the quantity and typical qualities of maternal care behaviors were indistinguishable in the two environments, their patterns and rhythms differed drastically and influenced emotional outcomes. Specifically, unpredictable, fragmented maternal care patterns translated into high-entropy rates of sensory signals to the offspring in the impoverished cages. During adolescence, these offspring had significant reductions in sucrose preference and in peer-play, two independent measures of the ability to experience pleasure. This adolescent anhedonia, often a harbinger of later depression, was not accompanied by measures of anxiety or helplessness. Dopaminergic pleasure circuits underlying anhedonia are engaged by predictable sequences of events, and predictable sensory signals during neonatal periods may be critical for their maturation. Conversely, unpredictability maternal-derived signals may disrupt these developmental processes, provoking anhedonia. In sum, high-entropy and fragmented patterns of maternal-derived sensory input to the developing brain predicts, and might promote, the development of anhedonia in rodents, with potential clinical implications.

## Introduction

Emotional disorders often commence during adolescence and stem from interactions between genes and environment, especially during sensitive developmental periods.^1, 2, 3, 4^ Indeed, limbic/emotional systems are not fully mature early in postnatal life, and their life-long function is influenced by environment-derived experiences,^1, 3^ similar to sensory systems.^5, 6^ Parental care is a principal source of environmental sensory signals to the developing brain.^7, 8^ Unsurprisingly, the contribution of maternal care to offspring outcome has been a topic of intense study in humans,^9, 10, 11^ primates^12, 13, 14, 15^ and rodents.^16, 17, 18, 19^ Indeed, a compelling body of work has linked the presence and quantity of maternal care behaviors^10, 11^ as well as several of their qualitative aspects^9, 20, 21^ to emotional outcome of children and experimental animals.^16, 17, 18, 19^

A key enigma is the identity of the specific aspects of maternal signals that are perceived by the developing brain and that influence the developing emotional networks, promoting healthy or aberrant emotional outcomes.^1, 3, 4, 16, 20^ To address this enigma, we applied mathematical analyses to maternal rodent behaviors in both control (CTL) and modified rearing environments and examined the emotional outcomes of adolescent offspring. To modulate maternal care behaviors, we used a naturalistic paradigm of impoverished rearing environment created by limiting the bedding and nesting materials in the cages (LBN) for 1 week.^22^ We examined, in addition to traditional measures of duration of maternal-nurturing behaviors and several qualitative aspects of care known to influence outcome,^23^ the patterns and sequences of maternal care. We focused on the predictability and fragmentation of these sources of sensory information to pups.

Adolescent males reared in the LBN cages had a reduced capacity to experience pleasure (anhedonia)^24^ and this was evident in two independent tests. Preference for sucrose and playing with peers, both dependent on normal function of the dopaminergic pleasure/reward system,^25, 26, 27, 28, 29, 30, 31^ were diminished in LBN-reared vs CTL adolescents. Unexpectedly, these anhedonic characteristics could not be explained by differences in the quantity of maternal contact or by several typical qualitative measures of maternal-nurturing behaviors, because these did not differ between the two groups of dams. Rather, novel analyses of predictability and fragmentation revealed higher entropy rates of maternal signals to groups of pups who developed abnormal pleasure/joy behaviors during adolescence. Because the pleasure/reward circuits are engaged by predictable sequences of events^32, 33^ and are immature during early postnatal life, we speculate that predictable sensory signals may be critical for the maturation of these circuits.^34, 35^ In contrast, unpredictability of early-life sensory signals may disrupt these developmental processes, provoking anhedonia.^36^ These findings suggest that high-entropy and fragmented patterns of maternal-derived sensory input to the developing brain predict the development of anhedonic behaviors during adolescence.

## Materials and methods

A complete description is found in the Supplementary Information, available on the Translational Psychiatry website (http://www.nature.com/tp).

All experiments were performed in accordance with the National Institutes of Health (NIH) guidelines on laboratory animal welfare and approved by the Institutional Animal Care and Use Committee.

### Animals

Subjects were born to primiparous time-pregnant Sprague-Dawley rat dams that were maintained in an uncrowded, quiet animal facility room on a 12 h light/dark cycle with *ad libitum* access to lab chow and water. Parturition was checked daily, and the day of birth was considered postnatal day 0 (P0). On P2, pups from several litters were gathered, and 12 pups (6 males and 6 females) were assigned at random to each dam, to obviate the potential confounding effects of genetic variables and of litter size. At P21, only male rats were kept and were housed two to three per cage in a quiet, uncrowded facility on a 12 h light/dark cycle, with *ad libitum* access to lab chow and water. Rats were subjected to the 2-week sucrose preference test at ~7 weeks of age and to the other behavior tests during the 2nd week of sucrose preference test.

### Manipulation of maternal care using limited bedding/nesting cages

The experimental paradigm involved rearing pups and dams in 'impoverished' cages as described previously.^22, 37, 38^ Briefly, routine plastic rat cages were fitted with a plastic-coated aluminum mesh platform sitting ~2.5 cm above the cage floor (allowing collection of droppings). Bedding was reduced to only cover cage floor sparsely, and one-half of a single paper towel was provided for nesting material. CTL dams and their litters resided in standard bedded cages, containing 0.33 cubic feet of cob bedding, which was also used for nest building. CTL and experimental cages were undisturbed during P2–P9, housed in a quiet room with strong laminar airflow, preventing ammonia accumulation. On P10 the experimental group was transferred to routine cages, where maternal behavior normalized within hours.^38, 39^

### Characterization and mathematical analyses of maternal behaviors

We analyzed several aspects of maternal behaviors. First, we compared the total amount of the individual nurturing behaviors described above (licking/grooming, nursing) in the CTL and LBN environments using four cohorts collected at different times. Each cohort consisted of two dams in CTL environments and two dams in LBN cages. We collected data during both the light and dark phase for three cohorts and during the light phase only for the fourth cohort.

Second, we compared the mean length of an individual licking/grooming bout (and the number of bouts) for mothers in the two groups. Fragmentation of maternal care would be expected to result in more frequent and shorter bouts.

Third, while fragmentation refers to characteristics of the maternal delivery of a particular type of care (for example, licking/grooming), unpredictability focuses on transitions from one type of maternal behavior to another.

### Assessments of anhedonia- and depressive-like behaviors

As measures of the spectrum of depressive-like behaviors, we analyzed sucrose consumption, considered a measure of anhedonia,^40^ social interaction (especially play behavior), which is a powerful reward to young rodents activating brain reward pathways,^31, 41^ and the immobility time in the forced swim test, considered a measure of 'behavioral despair'. All measurements and analyses were carried out without knowledge of treatment group.

### Analysis of anxiety-like behaviors

As measures of anxiety, we analyzed exploration times in the open field apparatus as well as time on the open arms in the elevated plus maze. A computerized video tracking system EthoVision (Noldus Information Technology, Wageningen, The Netherlands) was used to calculate the time spent in the inner 'anxiogenic' regions of the apparatus. Both tests were conducted in a quiet, empty and dimly lit room with no visual cues to distract the tested rat. Anxiety-like behaviors were conducted on a separate cohort from that used for the depressive-like behavior and a single test per day was run to avoid the potential confounding effect of performing two consecutive tests on a single rat. The rats were subjected to the elevated plus maze test and 24 h later to the open field test. All data collection and analyses were carried out without knowledge of treatment group.

### Statistical analyses

The early-life environment effects were assessed using a variety of statistical tests. Differences in sucrose consumption were assessed using a two-way repeated-measures analysis of variance (RM-ANOVA). Differences in social interaction behaviors, forced swim, elevated plus maze and open field tests were analyzed by Student's *t*-tests with Welch's corrections when necessary. Two-way RM-ANOVA and Student's *t*-tests were used for comparisons of maternal behavior measures. All ANOVAs were followed by Bonferroni's *post hoc* multiple comparisons test. Mean duration of individual licking/grooming bouts and entropy rates were carried out via *t*-tests for the group membership coefficient in a linear model that allows for cohort effects.

## Results

### Preference for sucrose and playing with peers, two independent measures of pleasure vs anhedonia, are influenced in adolescent rats by early-life experience

**Figure 1:**
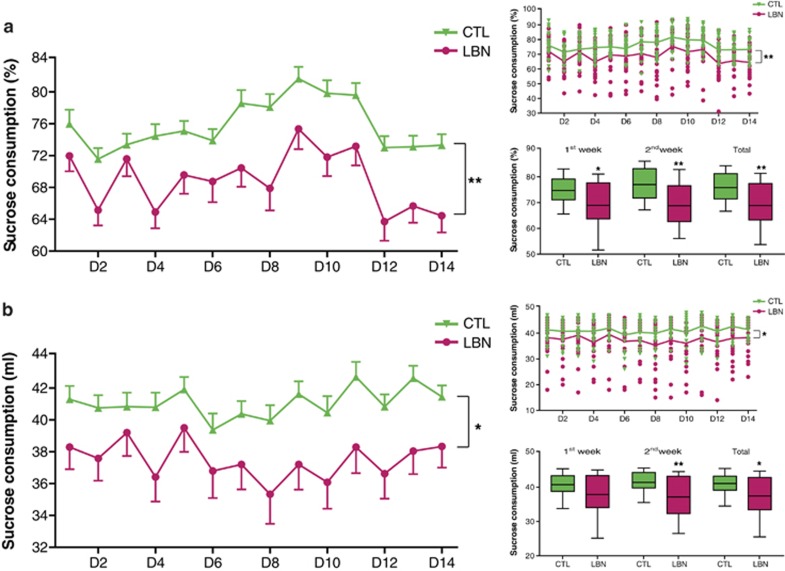
Sucrose consumption, a measure of anhedonia, is reduced in adolescent male rats reared in cages with limited bedding/nesting material (LBN). (**a**) Relative consumption of sucrose (% of total liquid intake) as well as (**b**) sucrose consumption (ml), measures of pleasure/reward seeking, were reduced over a 2-week period in individual rats reared in LBN cages during a sensitive early postnatal period as compared with those reared in routine laboratory cages (CTL) (*n*=24 per group). Data are also presented as daily consumption for individual rats as well as box and whisker plot (10th and 90th percentiles) for the groups. Horizontal bars represent mean values. Black asterisks denote statistical significance using the two-way RM-ANOVA; *P<0.05; **P<0.01. RM-ANOVA, repeated-measures analysis of variance.

To modulate dam-nurturing behaviors, we used a naturalistic paradigm of impoverished rearing environment based on LBN.^19, 22, 37, 38, 39^ Male rats reared for a week in LBN cages were of normal weight in late adolescence/young adulthood (postnatal days 50–60). However, sucrose consumption of these rats (*n*=24 per group) over a 2-week period was significantly reduced compared with age-matched peers raised in routine cage environment (CTL) (Figure 1). The relative consumption of sucrose and water (% Sucrose, a measure of preference for a sweet taste) (F_(1,46)_=9.04; *P*=0.004; two-way RM-ANOVA, Figure 1a) as well as total sucrose consumption were significantly affected by the early-life experience (F_(1,46)_=5.29; *P*=0.026; two-way RM-ANOVA, Figure 1b). Overall fluid consumption (sum of water and sucrose solution) did not differ between the groups (F_(1,46)_=0.0019; *P*=0.966; two-way RM-ANOVA, Supplementary Figure 1). Reduced relative and absolute sucrose consumption is considered a measure of anhedonia,^40^ which is often a herald of depression.^42, 43^

**Figure 2:**
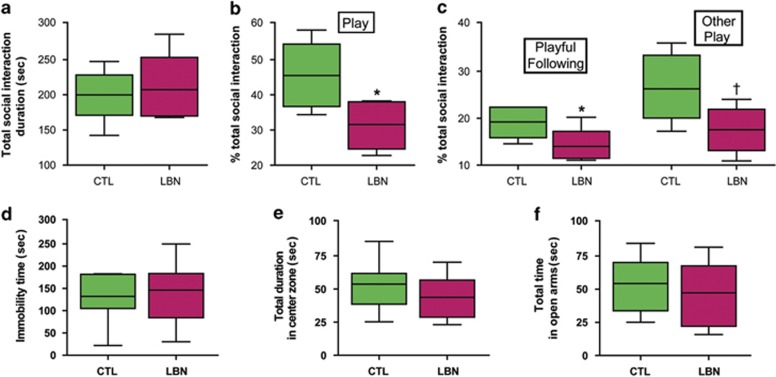
Peer-play, a second measure of pleasure, is significantly altered by the rearing environments. (**a**) Total duration of social interactions was not different in rats reared in limited bedding/nesting material (LBN) vs control cages. However, (**b**) the fraction of total time spent playing with peers, a measure of pleasure or joy, as well as (**c**) time spent in following or other play behaviors were significantly lower in these adolescent males vs the controls (*P*<0.05, Student's *t*-tests; *n*=5 per group). (**d**) Immobility time in the Porsolt forced swim test, a measure of learned helplessness, did not differ significantly between groups. In two measures of anxiety-like behaviors, (**e**) open field and (**f**) elevated plus maze, durations of behaviors considered indicative of anxiety did not differ significantly among groups (all *P*>0.05, Student's *t*-tests; values are provided in seconds; *n*=12 per group for (**d**–**f**). Data presented as box and whisker plot show the 10th and 90th percentiles. Horizontal bars represent mean values. Black asterisks denote statistical significance using the Student's *t*-test; *P<0.05, ^†^*P*=0.056. CTL, control.

To further probe if the reduced preference for sucrose reflected diminished seeking of stimuli considered pleasurable or joyful, we employed a second dopamine-dependent measure, namely peer-play during social interaction.^30, 31, 41^ We measured both total duration of engagement with peers, as well as specific behaviors. We focused on peer-play, a group of social interactions that are considered to denote joy and pleasure, and distinguished them from sniffing and other non-play behaviors.^44, 45, 46^ The overall duration of social interactions during the 10-min video-observation period did not distinguish between adolescent rats reared in LBN cages and those from CTL cages (*n*=5 per group; *t*_8_=0.253, *P*=0.807; Figure 2a). However, the fraction of time devoted to playing with peers, including following, chasing and other play behaviors (see Supplementary Methods), was significantly lower in the group reared in LBN cages (31% vs 45% *t*_8_=2.677, *P*=0.028; student's *t*-test, Figures 2b and c). Reduced social play in rodents is generally considered to reflect diminished joy and pleasure.^31, 41, 44^

The anhedonic phenotype of the adolescent LBN-reared rats was relatively selective, because these rats did not differ significantly from CTLs in an additional test of depressive-like behavior, the Porsolt forced swim test.^47^ Immobility time, the outcome measure of this test, was comparable in both groups (*n*=12 per group; LBN: 145.8±19.9 s, CTL: 131.4±15.9 s; *t*_22_=0.567, *P*=0.576, Student's *t*-test, Figure 2d). In addition, the LBN-reared rats did not appear to be anxious or fearful on two independent rodent tests considered sensitive to these traits. Time in the center zone of the open field arena did not differ significantly between groups (*n*=12 per group; LBN: 43.5±4.7 s, CTL: 53.6±5.6 s; *t*_22_=1.38, *P*=0.181, Student's *t*-test, Figure 2e). Similar results were obtained in the elevated plus maze, where the duration of inhabiting the brightly lit arm did not distinguish between groups (LBN: 47.2±7.3 s, CTL: 54.4±5.8 s; *t*_22_=0.772, *P*=0.449, Student's *t*-test, Figure 2f). Together, these findings suggested deficits in the dopamine-dependent pleasure/reward circuit, with only modest trends for anxiety and depression-like behaviors, in adolescent rats exposed to the LBN experience.

The appearance of anhedonic behaviors during adolescence in humans is important and ominous because it is considered a source of risk-taking behavior and addiction and often heralds depression.^24, 42, 43^ Therefore, we investigated the potential basis of these behaviors. The manipulation of early-life environment employed here did not provoke growth retardation or poor weight gain (*t*_39.56_=1.456, *P*=0.153, Student's *t*-test, Supplementary Figure 2). Hence, we reasoned that these disturbances might be a result of an altered repertoire of maternal-nurturing behaviors that were received and perceived by the developing pups.^20, 39, 48^

### Quantitative and classical qualitative measures of maternal-nurturing behaviors do not predict offspring outcome

**Figure 3:**
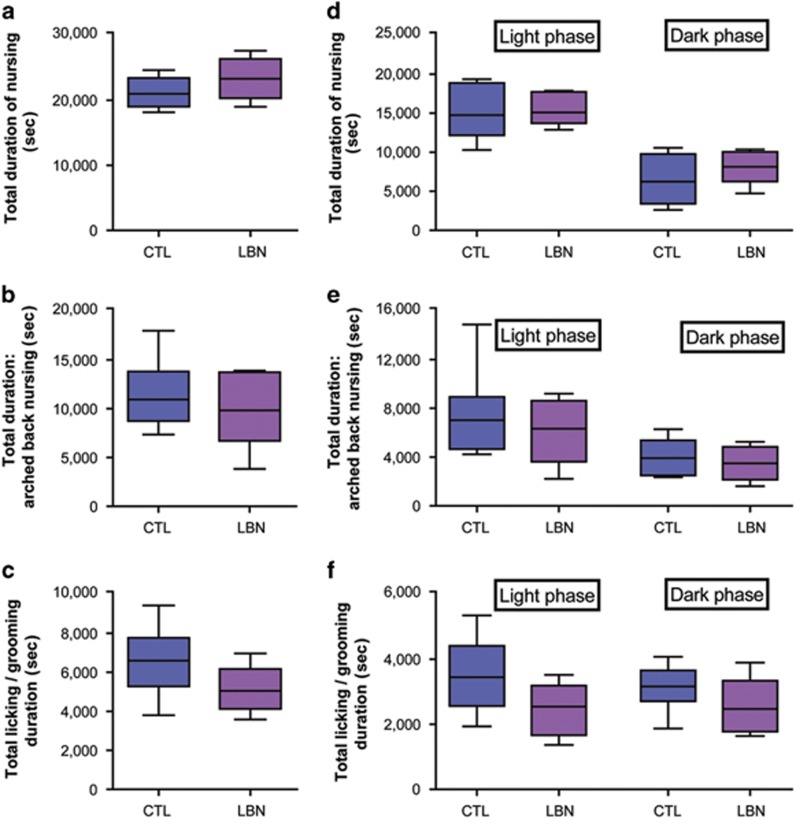
Several measures of the quantity and quality of maternal care do not distinguish dams in routine cage environment (CTL) from those in limited bedding/nesting (LBN) cages. (**a**) Total duration of nursing was not significantly different. (**b**) Durations of arched-back nursing, considered a measure of optimal quality of maternal care, were similar between groups. (**c**) Duration of time spent licking/grooming pups were comparable in CTL and LBN dams (all *P*>0.05, Student's *t*-tests). When separate analyses were performed for nurturing behaviors during the light phase or the dark phase, (**d**) nursing, (**e**) arched-back nursing and (**f**) licking/grooming times did not differ between groups (all *P*>0.05, Bonferroni's *post hoc* test). Values are provided in seconds, and are sum of observations over two 50-min periods per day for 8 days. (*n*=6 per group). Data presented as box and whisker plot show the 10th and 90th percentiles. Horizontal bars represent mean values. CTL, control.

To test this directly, we mathematically analyzed maternal care. Total durations of several types of nurturing behaviors did not differ among groups (Figure 3). Specifically, total maternal contact during nursing (Figure 3a), arched-back nursing (Figure 3b) and licking/grooming times (Figure 3c) were similar in dams within LBN cages and dams housed in standard cages (for contact/nursing: 23 290±1299 vs 21 050±995.7 s; for arched-back nursing: 9842±1580 vs 10 933±1560 s; for licking/grooming: 5034±497.4 vs 6606±755 s; all *P*>0.05 Student's *t*-test, Figures 3a and c). Because rodents are nocturnal and aberrant maternal care patterns might emerge selectively at night, we analyzed dams' nurturing behaviors separately during the light and dark phases (Figures 3d and f). During the dark phase, both CTL dams and dams housed in LBN cages spent less time nursing (Figure 3d, F_(1,10)_=30.32; *P*<0.001) including arched-back nursing (Figure 3e, F_(1,10)_=8.538; *P*=0.015; two-way RM-ANOVA) compared with the light phase. However, durations of these nurturing behaviors did not differ between groups (all *P*>0.05, Bonferroni's *post hoc* test). The cage environment did not influence light phase or dark phase nursing (Figure 3d), arched-back nursing (Figure 3e) and/or licking/grooming durations (Figure 3f, F_(1,10)_=1.867; *P*=0.202, F_(1,10)_=0.241; *P*=0.634, F_(1,10)_=3.019; *P*=0.113, respectively; two-way RM-ANOVA). These findings suggested that neither quantity of maternal care nor several qualitative aspects of dam behavior known to influence outcome^23^ accounted for the anhedonia-like behavior in late adolescents males reared in the modified cages. We then analyzed the patterns and sequences of maternal care in LBN cages in comparison with the routine cages, and found profound differences.

### Unpredictability and fragmentation of maternal care patterns predict offspring adolescent outcome

**Figure 4:**
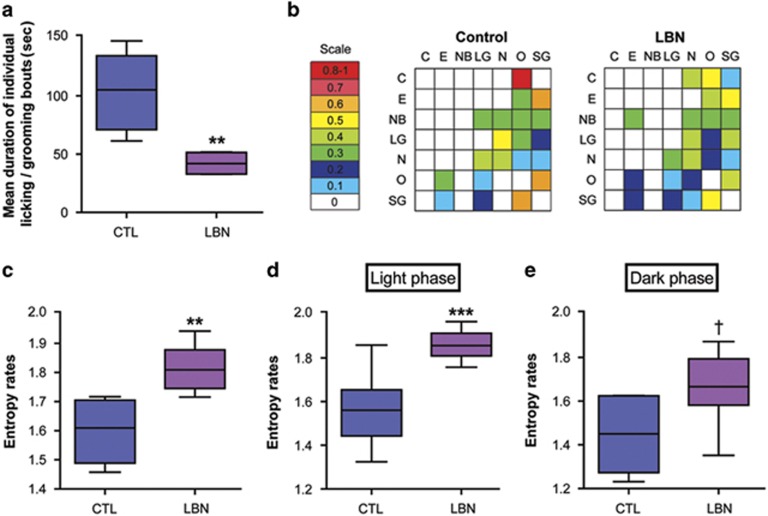
Fragmentation, unpredictability and high-entropy rates of maternal care in limited bedding/nesting (LBN) cages. (**a**) Mean duration of individual bouts of licking/grooming was drastically shorter in the LBN group, creating a fragmented sensory input to the pups. (**b**) Heat-map depicting behavioral sequences of an individual dam. The probability that a given behavior will follow another is depicted along a color scale. Controls tended to have either high (orange–red) or very low (white and light blue) probabilities of behavioral sequences. In contrast, LBN mothers had mid-range probabilities (bright blues and greens) to engage in any sequence of nurturing behaviors, suggesting that these sequences were random. (**c**) Entropy was employed to mathematically define the unpredictability of maternal behavior patterns (see Materials and methods). Entropy rates in LBN dams were significantly higher on average than those of CTL dams. (**d** and **e**) When broken down for entropy rates during light phase and dark phase, the group differences persisted, with a stronger effect during the light phase period. Data presented as box and whisker plot show the 10th and 90th percentiles. Horizontal bars represent mean values. Black asterisks denote statistical significance using a linear model; ***P<0.001, **P<0.01, ^†^*P*=0.08. CTL, control.

The mean duration of individual licking/grooming bouts for LBN dams (42 s) was significantly shorter than mean bout length for the CTLs (105 s), even accounting for cohort effects (*t*_8_=4.781, *P*<0.01, linear model, Figure 4a). These shortened durations of individual bouts indicated a fragmentation of the sensory signals received by the pups. To assess the consistency and predictability of the maternally derived sensory signals, we used a matrix of conditional probabilities that a given maternal behavior 'A' is followed by a second behavior 'B'. High values for some of these values suggest predictable sequences. As shown in the heat-map (Figure 4b), CTL dams tended to have either high (orange/red) or low (white and light blue) probabilities of behavioral sequences, whereas LBN mothers had mid-range probabilities to engage in any sequence of nurturing behaviors (bright blue and green), suggesting that these sequences were more random. Accordingly, the mean entropy rate for LBN maternal behavior (1.81) was significantly higher than for the CTL group (1.61; even after accounting for cohort effects; *t*_8_=−5.081, *P*<0.01, linear model, Figure 4c). These differences were apparent both during the light phase (*n*=8 per group) and during the dark phase (*n*=6 per group) with the effects considerably stronger in the light phase (Figures 4d and e).

The average number of individual licking/grooming bouts was significantly higher in the LBN dams: 123.5 per total observation period, compared with 67.5 for the CTLs. The number of bouts was correlated with the entropy rates (*r*=0.66), as was mean duration of individual licking/grooming bouts (*r*=−0.42). Broken down within each group, there were no consistent correlations between entropy and the duration or the numbers of behavioral bouts in the CTLs. However, for dams housed in the LBN cages, there was a consistent (both in light phase and dark phase) negative correlation between entropy and mean bout length. Thus, in the LBN group, a high-entropy rate and a high degree of fragmentation tended to coexist in the same dam.

## Discussion

The principal findings of these studies are that even with equal quantities and similar repertoires of maternal-nurturing behaviors in CTL and LBN cages, the emotional outcomes of adolescent male offspring differed drastically. While several elements distinguished the two early-life environments, a major difference was the pattern of maternal behaviors (see below). The maternal behaviors evaluated here, especially licking and grooming, have been established as the key sources of active, maternally derived sensory input to the pups that contribute powerfully to a number of offspring outcome measures (see below).^49, 50^ In the LBN cages, unpredictable behavioral sequences generating high-entropy rates, coupled with fragmented, short-duration bouts of individual caring behaviors. These were then predictive of anhedonia-like characteristics of adolescent offspring.

Maternal care has been well-recognized as influencing offspring outcome. In addition, several groups have demonstrated that passive maternal contact^50^ and the nutrition received via nursing^51^ control peripheral components of the hypothalamic-pituitary-adrenal system. However, gene expression within the brain and the long-lasting emotional and cognitive consequences of early-life experience are governed primarily by sensory signals derived from active maternal-nurturing behaviors.^16, 19, 20, 23, 34, 49, 50^ Indeed, a number of comprehensive studies have manipulated the quantity of maternal care using well-established models of maternal separation.^52, 53, 54, 55, 56, 57, 58, 59^ Reduced quantity of maternal care through intermittent deprivation has been reported to increase,^55, 57^ reduce^52, 55^ or not change^54, 59^ sucrose preference. In addition, reduced quantity of maternal care has often led in the offspring to measures of depressive-like behavior in the forced swim test,^52, 60, 61^ as well as to anxiety-like behaviors.^61, 62^ These behavioral deficits were not significantly altered by the LBN-cage early-life experience. In addition, in the current studies, the quantity of maternal care did not differ among the LBN and routine rearing environments. While it is conceivable that subtle unrecognized changes of the quantity of maternal care took place in the LBN cages, we think such changes are unlikely to be a cause of the adolescent outcomes, because even the major quantitative changes of maternal care induced by intermittent maternal separation did not lead to the disruption of the pleasure/reward system that was found here.

In addition to altered patterns of maternal care, other differences that existed between the experiences in CTL and LBN cages might account for the divergent outcomes.^63^ For example, the LBN in the cages provoke stress in the pups, and early-life stress is known to alter a number of cognitive and emotional outcome measures later in life. However, while stress is generated both in pups exposed to the LBN experience^37^ and in those exposed to maternal deprivation,^64^ a reduced capacity to experience pleasure from sucrose or peer-play did not emerge after early-life stress induced by maternal deprivation.^57, 59^ Therefore, stress generated by the limited bedding environment is unlikely to be a sole or major source of the deficits in pleasure/joy-like behaviors generated by the LBN-cage experience. As total durations of both nursing (passive) and of active maternal care did not differ in the two conditions, we believe that a key feature distinguishing early-life experience in CTL and LBN cages stems from the distinctive patterns of maternal-derived sensory inputs received by the two groups during a critical developmental period.^1, 3, 5, 6, 11, 20^ Specifically, sensory input from the mother arrived in short fragmented bouts in the LBN group, and in longer bouts in the CTLs. In addition, sequences of behaviors were significantly more predictable in the CTL cages, so that entropy rates, a measure of randomness and unpredictability, were higher in the LBN group. Because the behaviors of dams with high-entropy patterns also tended to be more fragmented, the sensory experiences of neonatal rats reared by these dams were truly chaotic.^65^

How might unpredictable sensory input early in life influence sucrose preference and social play during adolescence? Both of these behaviors depend on an intact dopaminergic pleasure/reward system.^25, 26, 27, 28, 29, 30, 31^ The dopaminergic reward system is not fully mature until the 3rd postnatal week in rodents^35^ and is sensitive to the influence of early-life experiences.^34, 36^ Importantly, predictable sequences of events have been reported to engage the reward system.^32, 33^ These observations lead us to speculate that 'predictable' patterns of maternal care provide crucial cues for maturation of the pleasure/reward system.^6, 32, 34^ In the absence of such input, the ability to experience reward from pleasurable sensations including the sweetness of sucrose or the joy of playing with peers, might be impaired^34, 36^ manifesting as anhedonia.^43^

Sensory input early in life governs neuronal activity, which influences brain organization,^5, 6, 66^ as demonstrated for visual^5^, tactile^6^ and olfactory^67^ sensory systems. In analogy, we speculate that patterns of maternal-derived sensory input, specifically unpredictable and fragmented patterns, might influence the maturation of emotional systems within the developing brain. While the mechanisms for this speculated process require further study, its implications are profound: identifying optimal nurturing environments for emotional outcome may help reduce the high and increasing prevalence of emotional problems during adolescence.^68^
