# Supplementary material for: Fragmentation and high entropy of neonatal experience predict adolescent emotional outcome
Source: Transl Psychiatry. 2016 Jan 5;6(1):e702–. doi: 10.1038/tp.2015.200 (PMC5068874; doi:10.1038/tp.2015.200)
Supplement: Supplementary Information [file tp2015200x1.doc]

**Supplementary Information:**

**Supplementary Material and methods:** Complete description

**Supplementary figure legends:**

Supplementary Figure 1: The total volume of liquid consumed is not altered by rearing environment.

Supplementary Figure 2: Body weight during adolescence is not consistently influenced by rearing environment.

**Supplementary Material and methods**

All experiments were performed in accordance with the National Institutes of Health (NIH) guidelines on laboratory animal welfare and approved by the Institutional Animal Care and Use Committee.

**Animals**

Subjects were born to primiparous time-pregnant Sprague-Dawley rat dams that were maintained in an uncrowded, quiet animal facility room on a 12 h light/dark cycle with *ad libitum* access to lab chow and water. Parturition was checked daily, and the day of birth was considered postnatal day 0 (P0). On P2, pups from several litters were gathered, and 12 pups (6 males and 6 females) were assigned at random to each dam, to obviate the potential confounding effects of genetic variables and of litter size. At P21, only male rats were kept and were housed 2-3 per cage in a quiet, uncrowded facility on a 12 h light/dark cycle, with *ad libitum* access to lab chow and water. Rats were subjected to the sucrose preference test at around 7 weeks of age and to the other behavioral tests during the second week of the two-week sucrose preference test.

**Manipulation of maternal care using limited bedding/nesting cages**

The experimental paradigm involved rearing pups and dams in “impoverished” cages as described previously.[^1-3^](#_ENREF_1) Briefly, routine plastic rat cages were fitted with a plastic-coated aluminum mesh platform sitting ~2.5 cm above the cage floor (allowing collection of droppings). Bedding was reduced to only cover cage floor sparsely, and one-half of a single paper towel was provided for nesting material. Control dams and their litters resided in standard bedded cages, containing 0.33 cubic feet of cob bedding, which was also used for nest building. Control and experimental cages were undisturbed during P2–P9, housed in a quiet room with strong laminar airflow, preventing ammonia accumulation. On P10 the experimental group was transferred to routine cages, where maternal behavior normalized within hours.

Maternal behaviors were assessed through direct and continuous observation of cages for 50 minute periods each twice a day. Mothers were identified as performing one of 7 different behaviors: licking/grooming pups, carrying pups, eating, nursing (in high-or low-arched posture), nest building, off pups, or self-grooming. Coding of maternal behaviors was highly reliable as established by repeated coding by four different individuals (JM, XZ, YTM and LR).

**Characterization and mathematical analyses of maternal behaviors**

We analyzed several aspects of maternal behaviors. First, we compared the total amount of the individual nurturing behaviors described above (licking/grooming, nursing) in the control (CTL) and limited-bedding/nesting (LBN) environments using four cohorts collected at different times. Each cohort consisted of two dams in control environments and two dams in LBN cages. We collected data during both the light and dark phase for three cohorts and during the light phase only for the 4^th^ cohort.

Second, we compared the mean length of an individual licking/grooming bout (and the number of bouts) for mothers in the two groups. Fragmentation of maternal care would be expected to result in more frequent and shorter bouts.

Third, whereas fragmentation refers to characteristics of the maternal delivery of a particular type of care (e.g. licking/grooming) unpredictability focuses on transitions from one type of maternal behavior to another. We derived a measure of unpredictably in the dams by focusing on the conditional probabilities that describe transitions in maternal behavior. To do this, we characterized the dam’s behavior using an empirical transition matrix <p_ij_> _i,j=1...M_ of conditional probabilities of moving from one behavior to another. The value p_ij_ measures the proportion of times, the dam was recorded as transitioning from behavior *i* to behavior *j* and M=7 is the total number of behaviors considered. Each row of the transition matrix corresponds to a single “initial” behavior or starting state, and each column to the behavior being transitioned into. Each row will sum to one,
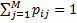
, reflecting the fact that there is always a transition to another behavior. As defined here
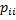
 = 0 because it is not possible to transition from a behavior to the same behavior. Note that alternative definitions of the transition matrix that allow for transitions from a behavior to itself are possible by discretizing time and assigning a single behavior to each time period. Such a definition would combine both fragmentation and unpredictability in a single measure.

Entropy is a natural summary measure of randomness or unpredictability of a distribution.[^5^](#_ENREF_5) There are a number of related measures that carry the term entropy. We focused here on the Shannon entropy. Values are ranged from zero (when the distribution is focused on a single outcome and thus perfectly predictable) to the logarithm of the number of possible states (when the distribution is spread equally among all possible outcomes and hence maximally unpredictable). Our focus on the transition matrix presents an extra level of complexity in the entropy calculation. Each row of the transition matrix represents a probability distribution, so entropy is calculated for each row and then combined for a single measure. To combine the row entropy values, we first calculated the stationary distribution
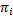
 of the Markov chain (which gives the long-term probability of the dam exhibiting each behavior). The stationary probabilities serve as weights for the entropy values, which produce a measure known as the entropy rate.[^5^](#_ENREF_5) We calculated the entropy rate of each dam from the transition matrix as follows:
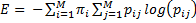
.

**Assessments of anhedonia- and depressive-like behaviors**

As measures of the spectrum of depressive-like behaviors we analyzed sucrose consumption, considered a measure of anhedonia,[^6^](#_ENREF_6) social interaction (especially play behavior) which is a powerful reward to young rodents activating brain reward pathways, and the immobility time in the forced swim test, considered a measure of “behavioral despair”. All measurements and analyses were carried out without knowledge of treatment group.

Sucrose preference test: Among the several methods for assessing sucrose preference, we employed the more chronic version, which has been extensively used as a measure of anhedonia.[^9-13^](#_ENREF_9) We used typical sucrose concentrations (1.5%,[^14^](#_ENREF_14) with a limited daily access (50 ml)) to avoid metabolic changes. The latter are induced with much higher sucrose concentrations during a longer period of exposure (e.g., 30% sucrose for 4 weeks).[^15^](#_ENREF_15) We assessed the consumption of the sucrose solution in comparison to water in a two bottle choice setting. Rats were individually housed for 7 days with ad libitum food, and given free access to two graduated bottles containing 50 ml of water. On day 8, rats were provided with 2 bottles, one containing 50 ml of tap water and the other 50 ml of 1.5 % sucrose solution during a 2 week period. These drinking solutions were available *ad libitum*, and were refreshed and refilled every day. The left/right position of the bottles was interchanged daily to avoid effects from a side-bias. Fluid (water and sucrose) consumption was recorded each morning, correcting for evaporation/spillage. We compared total sucrose consumption as well as the relative consumption of sucrose and water (% sucrose), the water consumption and the total volume of fluid that was consumed. This test was carried out in two cohorts. There was no difference between the cohorts so the data were combined.

Social interaction and peer-play test: The social interaction/peer-play test consists of three sessions separated by 24 hours.[^16^](#_ENREF_16) We assessed social interaction behaviors in an open field box (43 x 43 cm) placed in a dimly lit room. The habituation sessions (days 1 and 2), lasted 10 min each. Rats were placed in one corner of an open field and were allowed to explore the box. The apparatus was cleaned with 70% ethanol after each trial. During the testing session on day 3, rats were returned to the testing room, placed in the experimental apparatus and a younger test partner (to avoid aggressive behaviors) was placed in the box at the opposite corner. Behaviors during a 10 min epoch were monitored using a video camera. The duration of the following behavioral patterns were observed and scored: Following/chasing; kicking (using hind-legs); boxing (pawing upright); wrestling (rolling over one another in rough-and-tumble play); pinning; pouncing (attempting to nose or rub the nape of the neck); sniffing/licking; crawling/climbing. These behavioral patterns were divided into those related to play (following/chasing, wrestling, pouncing, pinning, kicking/boxing) and behaviors unrelated to play (crawling and sniffing/licking).[^17^](#_ENREF_17) No aggressive behaviors were noted.

Forced swim test: The forced swim test[^18^](#_ENREF_18) consists of two sessions separated by 24 hours, in a room illuminated with dim lighting. The habituation session (Day 1), lasted 15 min. Rats were placed in a glass cylinder (20 cm in diameter and 60 cm high) containing water (23-25ºC) filled to a depth of 45 cm. The test session occurred 24 hours later, and rats were placed in the cylinder for 5 min. Behavior was monitored using a video camera. The duration of immobility was scored and served as indicator of depressive-like behaviors. Water was replaced and containers cleaned between trials. This test was carried out on a single cohort.

**Analysis of anxiety-like behaviors**

As measures of anxiety, we analyzed exploration times in the open field apparatus as well as time on the open arms in the elevated plus maze. A computerized video tracking system (Noldus Ethovision) was used to calculate the time spent in the inner “anxiogenic” regions of the apparatus. Both tests were conducted in a quiet, empty and dimly lit room with no visual cues to distract the tested rat. Anxiety-like behaviors were conducted on a separate cohort from that used for the depressive-like behavior and a single test per day was run to avoid the potential confound of performing two consecutive tests on a single rat. The rats were subjected to the elevated plus maze test and 24 hours later, to the open field test. All data collection and analyses were carried out without knowledge of treatment group.

Elevated plus maze test: The elevated plus maze test consisted of two open arms (50 x 10 cm) and two cross-wise closed arms (50 x 10 x 40 cm) with an open roof 50 cm above the floor. Rats were placed in the center of the maze, facing an enclosed arm at the start of the experiment. Each rat was allowed one 5-min trial in the maze, and the maze was wiped with 70% ethanol after each trial. The time on open arms was used as an index of anxiety-like behavior.

Open field test: The open field consisted of open field box (100 x 100 cm) with black opaque walls and floor. Rats were placed in one corner of the open field, facing the wall at the start of the experiment. Each rat was allowed one 10-min trial and the box was cleaned with 70% ethanol after each trial. Duration of time spent in the center of the open field was used as an index of anxiety-like behavior.

**Statistical analyses**

The early-life environment effects were assessed using a variety of statistical tests. Differences in sucrose consumption were assessed using a two-way repeated-measures analysis of variance (RM-ANOVA). Differences in social interaction behaviors, forced swim, elevated plus maze, and open field tests were analyzed by Student’s *t*-tests with Welch’s corrections when necessary. Two-way RM-ANOVA and Student’s *t*-tests were used for comparisons of maternal behavior measures. All ANOVAs were followed by Bonferroni’s post hoc multiple comparisons test. Mean duration of individual licking/grooming bouts and entropy rates were carried out via *t*-tests for the group membership coefficient in a linear model that allows for cohort effects.

**Supplementary figure legends:**

**Supplementary Figure 1: The total volume of liquid consumed is not altered by rearing environment.** (**a**) Total volume of liquid (water, sucrose solution) that was consumed was similar in late adolescent rats reared in control (CTL) and limited bedding/nesting (LBN) environments. (**b**) As expected, reduced sucrose consumption is reflected in increased water consumption in LBN compared to CTL rats. (n=24/group). Data presented as daily consumption. Black asterisks denote statistical significance using the two-way RM-ANOVA; * *P*<0.05.

**Supplementary Figure 2:** **Body weight during adolescence is not consistently influenced by rearing environment.** Body weight was comparable in rats reared in limited bedding/nesting cages (LBN) and control (CTL) rats (*P*>0.05, Student’s *t*-test; n=24/group). Data presented as box and whisker plot show the 10^th^ and 90^th^ percentiles. Horizontal bars represent mean values.

1. Brunson KL, Kramar E, Lin B, Chen Y, Colgin LL, Yanagihara TK *et al.* Mechanisms of late-onset cognitive decline after early-life stress. *J Neurosci* 2005; **25:** 9328-9338.

2. Ivy AS, Rex CS, Chen Y, Dube C, Maras PM, Grigoriadis DE *et al.* Hippocampal dysfunction and cognitive impairments provoked by chronic early-life stress involve excessive activation of CRH receptors. *J Neurosci* 2010; **30:** 13005-13015.

3. Molet J, Maras PM, Avishai-Eliner S, Baram TZ. Naturalistic rodent models of chronic early-life stress. *Dev Psychobiol* 2014; **56:** 1675-1688.

4. Ivy AS, Brunson KL, Sandman C, Baram TZ. Dysfunctional nurturing behavior in rat dams with limited access to nesting material: a clinically relevant model for early-life stress. *Neuroscience* 2008; **154:** 1132-1142.

5. Cover T, Thomas J. *Elements of Information Theory*. 2nd edn. Wiley: New York, US, 2006.

6. Papp M, Willner P, Muscat R. An animal model of anhedonia: attenuation of sucrose consumption and place preference conditioning by chronic unpredictable mild stress. *Psychopharmacology (Berl)* 1991; **104:** 255-259.

7. Panksepp J, Siviy S, Normansell L. The psychobiology of play: theoretical and methodological perspectives. *Neurosci Biobehav Rev* 1984; **8:** 465-492.

8. Vanderschuren LJ, Niesink RJ, Van Ree JM. The neurobiology of social play behavior in rats. *Neurosci Biobehav Rev* 1997; **21:** 309-326.

9. Andre J, Zeau B, Pohl M, Cesselin F, Benoliel JJ, Becker C. Involvement of cholecystokininergic systems in anxiety-induced hyperalgesia in male rats: behavioral and biochemical studies. *J Neurosci* 2005; **25:** 7896-7904.

10. Barnes SA, Der-Avakian A, Markou A. Anhedonia, avolition, and anticipatory deficits: assessments in animals with relevance to the negative symptoms of schizophrenia. *Eur Neuropsychopharmacol* 2014; **24:** 744-758.

11. Becker C, Zeau B, Rivat C, Blugeot A, Hamon M, Benoliel JJ. Repeated social defeat-induced depression-like behavioral and biological alterations in rats: involvement of cholecystokinin. *Mol Psychiatry* 2008; **13:** 1079-1092.

12. Blugeot A, Rivat C, Bouvier E, Molet J, Mouchard A, Zeau B *et al.* Vulnerability to depression: from brain neuroplasticity to identification of biomarkers. *J Neurosci* 2011; **31:** 12889-12899.

13. Levine AS, Kotz CM, Gosnell BA. Sugars: hedonic aspects, neuroregulation, and energy balance. *Am J Clin Nutr* 2003; **78:** 834S-842S.

14. Overstreet DH. Modeling depression in animal models. In: Kobeissy FH (ed). *Psychiatric Disorders: Methods and Protocols. In: Methods in Molecular Biology.*, vol. 829. HumanaPress: New York, USA, 2012, pp 125–144.

15. Carvajal-Zarrabal O, Nolasco-Hipolito C, Aguilar-Uscanga MG, Melo-Santiesteban G, Hayward-Jones PM, Barradas-Dermitz DM. Avocado oil supplementation modifies cardiovascular risk profile markers in a rat model of sucrose-induced metabolic changes. *Dis Markers* 2014; **2014:** 386425.

16. File SE, Lippa AS, Beer B, Lippa MT. Animal tests of anxiety. *Curr Protoc Neurosci* 2004; **Chapter 8:** Unit 8 3.

17. Mikulecka A, Subrt M, Parizkova M, Mares P, Kubova H. Consequences of early postnatal benzodiazepines exposure in rats. II. Social behavior. *Front Behav Neurosci* 2014; **8:** 169.

18. Porsolt RD, Le Pichon M, Jalfre M. Depression: a new animal model sensitive to antidepressant treatments. *Nature* 1977; **266:** 730-732.
