# Supplementary figures and images for: Fragmentation and high entropy of neonatal experience predict adolescent emotional outcome
Source: Transl Psychiatry. 2016 Jan 5;6(1):e702–. doi: 10.1038/tp.2015.200 (PMC5068874; doi:10.1038/tp.2015.200)

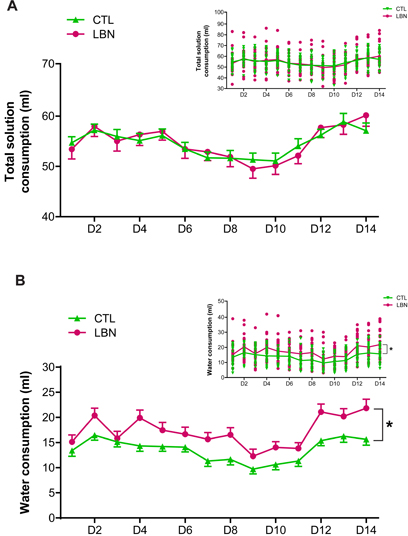

Supplement: Supplementary Figure 1 [file tp2015200x2.tif]

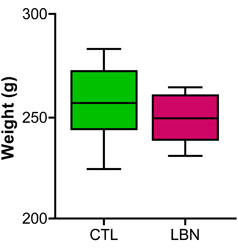

Supplement: Supplementary Figure 2 [file tp2015200x3.tif]
